# Long-term oncologic outcomes of natural orifice specimen extraction surgery versus conventional laparoscopic-assisted resection in the treatment of rectal cancer: a propensity-score matching study

**DOI:** 10.1186/s12893-022-01737-2

**Published:** 2022-07-25

**Authors:** Zhengliang Li, Huan Xiong, Tianyu Qiao, Shuai Jiao, Yihao Zhu, Guiyu Wang, Xishan Wang, Qingchao Tang

**Affiliations:** 1 Department of Colorectal Surgery, The Second Affiliated Hospital of Harbin Medical University, Harbin, 150081 China; 2 Department of Colorectal Surgery, National Cancer Center, National Clinical Research Center for Cancer, Cancer Hospital, Chinese Academy of Medical Sciences and Peking Union Medical College, 17 Panjiayuan Nanli, Beijing, 100021 China

**Keywords:** Rectal cancer, Natural orifice specimen extraction surgery, Laparoscopic surgery, Survival

## Abstract

**Background:**

Natural orifice specimen extraction surgery (NOSES) has been increasingly applied in radical surgery of abdominal and pelvic organs, but it is still in the exploratory stage. There is insufficient evidence to prove its efficacy.

**Methods:**

From January 2013 to June 2017, a total of 351 patients diagnosed with rectal cancer were eventually included in this study. Patients who underwent NOSES were assigned to the NOSES group, while patients undergoing conventional laparoscopic assisted resection were assigned as to the LAP group. Propensity score matching was used to align clinicopathological features between the two groups.

**Results:**

From the perioperative data and postoperative follow-up results of both groups, patients in the NOSES group had less intraoperative bleeding (47.0 ± 60.4 ml vs 87.1 ± 101.2 ml, *P* = 0.011), shorter postoperative gastrointestinal recovery (50.7 ± 27.3 h vs 58.6 ± 28.5 h, *P* = 0.040), less postoperative analgesic use (36.8% vs 52.8%, *P* = 0.019), lower postoperative pain scores (P < 0.001), lower rate of postoperative complications (5.7% vs 15.5%, *P* = 0.020), more satisfaction with body image (P = 0.001) and cosmesis (P < 0.001) postoperatively. The NOSES group had a higher quality of life. Moreover, there was no significant difference in overall survival (OS) and disease-free survival (DFS) between the two groups.

**Conclusion:**

NOSES could be a safe and reliable technique for radical resection of rectal cancer, with better short-term outcomes than conventional laparoscopy, while long-term survival is not significantly different from that of conventional laparoscopic surgery.

**Supplementary Information:**

The online version contains supplementary material available at 10.1186/s12893-022-01737-2.

## Introduction

Colorectal cancer (CRC) is currently the third most deadly and commonly diagnosed cancer in the world [1, 2]. Despite advances in surgical techniques and treatment medications, the mortality rate of CRC patients remains high. [3, 4]. However, radical surgery is still the primary treatment for CRC patients [5, 6]. With the continuous improvement of the effectiveness of equipment, laparoscopic radical resection has become the most important treatment [7]. Although it greatly reduces the trauma of surgery, the patients still experience a range of physical and psychological trauma due to the abdominal incision used for specimen extraction. Incisions increase the risk of postoperative complications, pain and potential psychological stress [8]. Therefore, extensive efforts have been made to reduce the trauma.

Natural orifice specimen extraction surgery (NOSES) refers to the use of traditional laparoscopic instruments, transanal endoscopic microsurgery (TEM) or soft endoscopy to obtain specimens through natural orifice (vagina or anus) without the aid of abdominal auxiliary incision after abdominal operation [9, 10]. The classical NOSES for rectal cancer include laparoscopic lower rectal cancer resection with transanal specimen extraction (NOSES-I), laparoscopic middle rectal cancer resection with transanal specimen extraction (NOSES-II), laparoscopic middle rectal cancer resection with transvaginal specimen extraction (NOSES-III), laparoscopic upper rectal cancer resection with transanal specimen extraction (NOSES-IV), laparoscopic upper rectal cancer resection with transvaginal specimen extraction (NOSES-V)[11].

However, the safety of NOSES has been questioned by some scholars. In this study, we aimed to explore the advantages and disadvantages of NOSES in radical resection of rectal cancer by retrospectively comparing the two different surgical methods for rectal cancer in terms of outcomes and cost-effectiveness, in order to give some strong evidence for the application of NOSES.

## Methods

### Selection of enrolled patients

Patients who were diagnosed with rectal cancer and underwent radical resection in the Department of colorectal surgery at the Second Affiliated Hospital of Harbin Medical University from January 2013 to June 2017 were included in this study. All cases were considered consecutive and representative. Patients underwent NOSES were allocated to the NOSES group, and those underwent conventional laparoscopic resection were allocated to the LAP group. Both surgeries were thoroughly explained to the patients by the treatment team; the safety and feasibility were evaluated. The patients made the final decision and signed the surgical informed consent form after discussion with their treatment team. All surgeries were performed in our center, and all surgeons completed the learning curve for laparoscopic radical rectal cancer and NOSES surgery. All patients acknowledged that the investigators kept all their treatment-related information and used for scientific research. The investigators also pledged to keep information about the patients private and confidential; and that the collection of data would not have any impact on the optimal treatment. Patients signed informed consent forms in this regard. This study obtained approval from the Ethics Committee of the Second Affiliated Hospital of Harbin Medical University. All procedures performed in this study met with the ethical standards of our institution and were in accordance with the Declaration of Helsinki.

The inclusion and exclusion criteria were referenced to the previous study from our team [8]. The inclusion criteria were as follows: (1) Aged from 18 to 80 years old; (2) Histopathology confirmed diagnosis of rectal cancer; (3) Preoperative imaging scan (CT and MRI) suggested that T stage was within T3; (4) BMI < 35 kg/m^2^; (5) Obtain informed consent of patients. The exclusion criteria were as follows: (1) Contraindication to laparoscopic surgery; (2) Emergency surgery was performed due to acute bowel obstruction, perforation or bleeding; (3) Combination with primary malignancies of other organs; (4) Patients with ostomy for any reasons; (5) Patients with multiple primary colorectal cancer; (6) Patients received neoadjuvant radiotherapy or chemotherapy; (7) Patients with a history of cancer; (8) Incomplete information or loss to follow-up.

### Surgical procedure

Bowel preparation was performed with oral polyethylene glycol electrolyte solution 12 h before surgery. After anesthesia, the patient was placed in the truncal position and a pneumoperitoneum was established. One 10 mm trocar was inserted from the laparoscope, one 12 mm working trocar in the right lower quadrant, two 10 mm trocar in the left lower and right upper quadrants, and one 5 mm trocar in the left upper quadrant. First, the liver, gallbladder, stomach, spleen, omentum, colon, small intestine, rectum, and pelvis were sequentially examined. Then the location of the tumor is explored. The surgeon may use a right-handed surgical forceps and a left-handed examining forceps to determine the location and size of the tumor. After incising Toldt’s fascia, the inferior mesenteric artery and vein were fully exposed and dissected, and a low ligation was performed to preserve the left colic artery. The mesentery is cut using an ultrasonic scalpel to fully expose the rectal and sigmoid intestinal canal, which is disconnected at the pre-cut line of the intestinal canal below the tumor. Finally, the patients in the NOSES group and the LAP group underwent surgery with different digestive reconstruction and specimen removal methods.

In the LAP group, the specimen was removed through a minimally incision via a skin protector in the lower abdomen. Then end-to-end anastomosis of the sigmoid colon and rectum was performed. Finally, the intraperitoneal lavage was administered with sterile distilled water and physiological saline. And the incision and trocar ports were closed.

**Fig. 1:**
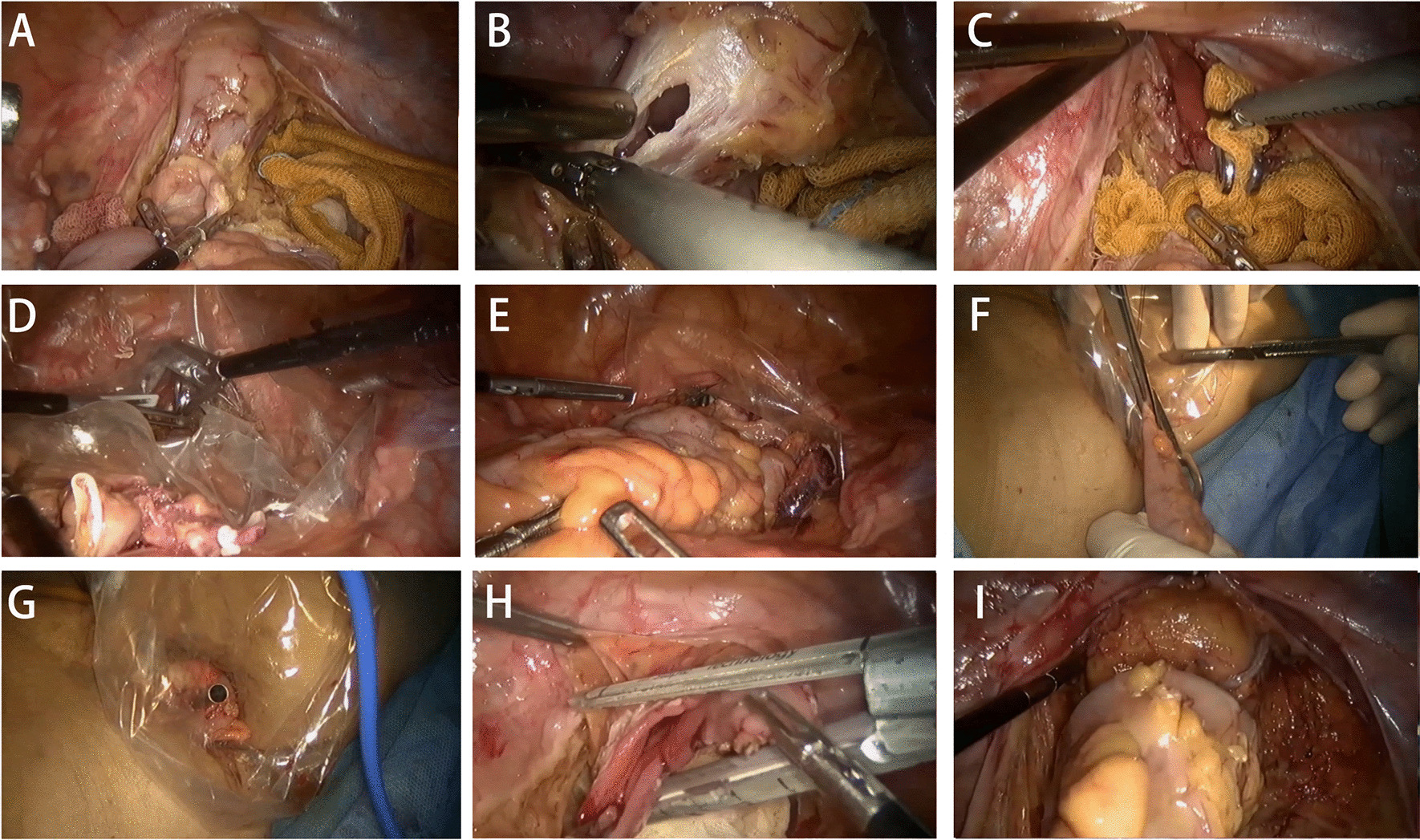
Disconnection and removal of specimens of the NOSES group. **A** The rectal mesentery is adequately naked by the surgeon. **B** The surgeon makes a transverse rectal incision 2 cm below the tumor. **C** The rectal stump is disinfected again. **D** Establish a sterile tumor-free channel. **E** The tumor-bearing rectum is closed and a protective sleeve is placed. **F** The tumor-bearing bowel is pulled out of the body through the protective sleeve and the specimen is removed. **G** The specimen is removed and the anastomotic staple holder is placed. **H** The distal incision is closed. **I** Complete the intestinal anastomosis

In the NOSES group, the NOSES procedure performed in median rectal cancer (NOSES-II) was taken as an example [12]. The plastic protective sleeve with one end ligated for backup was first placed through the main operating hole in the right lower abdomen, and the operator performed adequate nudity of the rectal mesentery (Fig. 1A). After anal dilatation, the perineal group assistant cleaned and flush the rectum using 1% povidone-iodine. The rectum is incised transversely 2 cm below the tumor (Fig. 1B). This can prevent residual povidone-iodine from entering the peritoneal cavity by pre-ligating the intestinal canal with band ligation. The perineal group assistant used oval forceps and iodine gauze to decontaminate the rectal stump again (Fig. 1C), followed by transanal pulling of the protective sleeve out of the body and unfolding to establish a sterile and tumor-free access (Fig. 1D). The tumor-bearing rectum was closed and then the protective sleeve was placed (Fig. 1E), and the perineal group assistant replaced the oval forceps, pulled the tumor-bearing bowel out of the body through the protective sleeve and removed the specimen (Fig. 1F). After removing the specimen, a circular cutter closure staple holder was placed at the upper incision margin (Fig. 1G). The proximal intestine was returned to the abdominal cavity and the protective sleeve was withdrawn from the anus, followed by closure of the lower incisional margin with a linear cutter closure (Fig. 1H). The end-to-end anastomosis was performed by placing the circumferential cutting closure through the anus to reach the position of the lower incisional margin and aligns it with the upper incisional margin staple seat ( Fig. 1I). We recommend the routine use of two drains in the vicinity of the pelvic anastomosis. Finally, the abdominal cavity was rinsed with sterile distilled water and saline, and the trocar pinhole was closed.

### Data collection and follow-up

All basic information and treatment related information of patients was obtained from the Second Hospital of Harbin Medical University’s electronic medical records system. On the first, third, and fifth postoperative days, we evaluated the patients' pain by using the visual analog scale (VAS). Body Image Questionnaire (BIQ, see Additional file 1) was used to assess patients’ satisfaction with the physical appearance and the appearance of the scar at one month after surgery [13]. PFDI-20 (Pelvic Floor Distress Inventory-20) was used to assess the impact of lower urinary tract, gastrointestinal and pelvic organ prolapse on the quality of life before surgery and at three months after surgery [14]. Wexner Incontinence Score comprehensively assessed the type and frequency of incontinence and the impact of incontinence on the patient's life [15]. Here, it was used to assess patient’s anal function at six months after surgery (Additional file 1: Table S1). The EORTC QLQ-C30 scale is the core scale used in the European Organization for Research and Treatment (EORTC) quality of life scale system to determine the quality of life of all cancer patients [16]. In the present study, we accordingly standardized the scores of the EORTC QLQ-C30 scales to assess the quality of life of patients at 3 months after surgery [17].

Patients were followed up every 3 months at the first 2 years after surgery and every 6 months at the subsequent 3 years until the patient died or the study was terminated. Patients were followed up through the outpatient clinic appointment, letter or telephone consultation. The median follow-up was 51 months in the LAP group and 53 months in the NOSES group. All patients were followed up to death or the end of the study in June 2020.

### Statistical analysis

To reduce the bias inherent in this study, an 1:1 propensity score matching (PSM) was performed based on baseline information between the two groups, including gender, age, BMI, ASA score, CEA, T stage, N stage, tumor size and preoperative PFDI-20 scores [8]. The logistic regression model was used to analyze the variables assignment based on the baseline data of 212 patients, and the matching tolerance was 0.01. The quantitative data were presented as mean ± SD and analyzed using Student’s t-test. For continuous data, we used a two-factor repeated measures ANOVA for analysis. The Chi-square test or Fisher's exact test was used to analyze the variance of the categorical variables. *P* < 0.05 was considered statistically significant.

## Results

### Comparison of baseline information between the two groups

Based on the large bias in the retrospective study, we performed PSM analysis to homogenize the two groups of patients. After PSM, there was no significant difference in the baseline information between two groups of patients (Table 1).

**Table 1:** Baseline information for two groups of patients

Characteristics	Before PSM				After PSM			
	LAP (N = 163)	NOSES (N = 188)	t/χ^2^	P value	LAP (N = 106)	NOSES (N = 106)	t/χ^2^	P value
Gender			6.463	0.011			0.302	0.583
Male	95 (58.3%)	104 (44.7%)			54 (50.9%)	50 (47.2%)		
Female	68 (41.7%)	84 (55.3%)			52 (49.1%)	56 (52.8%)		
Age (years)	59.9 ± 11.1	59.5 ± 11.5	0.341	0.734	60.4 ± 11.0	59.3 ± 11.8	0.702	0.483
BMI (kg / m^2^)	22.9 ± 3.1	22.1 ± 2.9	2.186	0.029	22.1 ± 2.8	22.4 ± 2.7	-0.771	0.442
ASA grade			1.062	0.303			1.759	0,185
I/II	132 (81.0%)	160 (85.1%)			93 (87.7%)	86 (81.1%)		
III	31 (19.0%)	28 (14.9%)			13 (12.3%)	20 (18.9%)		
Location of the tumor from the anus			13.603	0.001			3.940	0.139
< 5 cm	40 (24.5%)	72 (38.3%)			26 (24.5%)	32 (30.2%)		
5 ~ 10 cm	97 (59.5%)	75 (39.9%)			64 (60.4%)	50 (47.2%)		
10 ~ 15 cm	26 (16.0%)	41 (21.8%)			16 (15.1%)	24 (22.6%)		
Diameter of tumor			4.643	0.031			0.196	0.658
< 5 cm	106 (17.6%)	46 (15.4%)			74 (69.8%)	71 (67.0%)		
> 5 cm	57 (82.4%)	142 (84.6%)			32 (30.2%)	35 (33.0%)		
Preoperative CEA ^a^			2.520	0.112			0.147	0.701
Positive	21 (12.9%)	36 (19.1%)			15 (14.2%)	17 (16.0%)		
Negative	142 (87.1%)	152 (80.9%)			91 (85.8%)	89 (84.0%)		
T Stage			11.737	0.003			2.793	0.247
Tis/T1	31 (19.0%)	48 (25.5%)			21 (19.8%)	15 (14.2%)		
T2	25 (15.3%)	50 (26.6%)			19 (17.9%)	28 (26.4%)		
T3	107 (65.6%)	90 (47.9%)			66 (62.3%)	63 (59.4%)		
N Stage			2.729	0.099			0.097	0.755
N0	113 (69.3%)	145 (77.1%)			79 (74.5%)	77 (72.6%)		
N1/N2	50 (30.7%)	43 (22.9%)			27 (25.5%)	29 (27.4%)		
Preoperative PFDI-20 scores	7.53 ± 2.04	7.35 ± 1.64	0.910	0.364	7.35 ± 2.04	7.38 ± 1.69	− 0.110	0.813

Results in the table are presented as mean ± standard deviation or number (%). a: the cut-off value was considered to be 5 ng/ml

*LAP* conventional laparoscopic assisted resection group, *NOSES*, natural orifice specimen extraction surgery group, *PSM* propensity score matching, *BMI* body mass index, *ASA score* American Society of Anesthesiologists score, *CEA* carcinoembryonic antigen, *PFDI-20* Pelvic Floor Distress Inventory-20

### Comparison of short-term outcomes between two groups

**Fig. 2:**
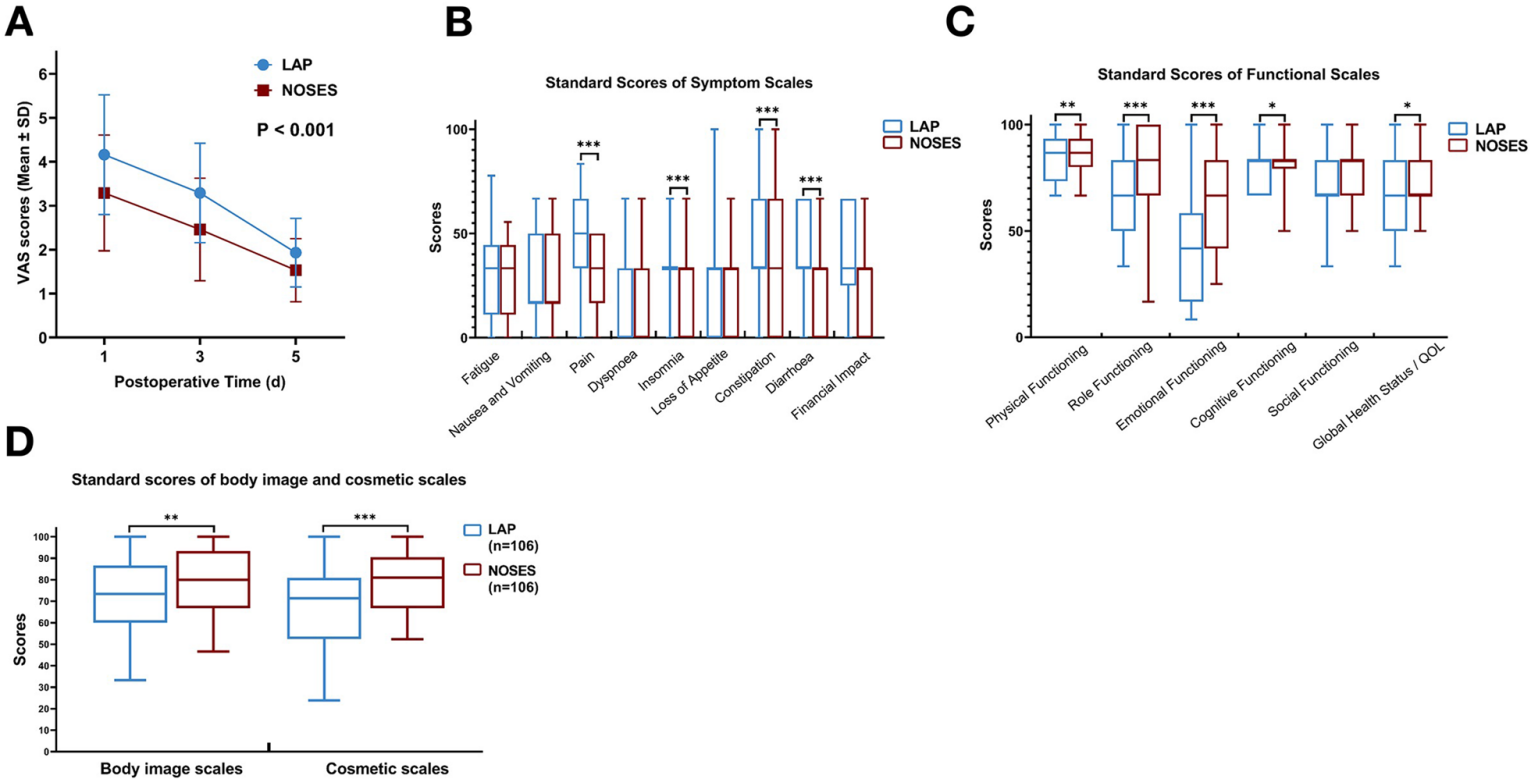
Comparison of short-term curative effect between two groups of patients. **A** Postoperative VAS scores in two groups of patients after PSM. **B**, **C** EORCT Quality of Life questionnaire-Core 30 results of two groups after PSM. B, Functional Scales. C: Symptom Scales. **D** Scores of body image and cosmetic scales after PSM. (Higher scores indicate better body image and higher satisfaction with scars). (*p < 0.05, **p < 0.01, ***p < 0.001)

Next, we use case statistics and follow-up information to compare the short-term outcomes of the two groups of patients. There is no significant difference in the operation time between the two groups, but the NOSES group was significantly better than the LAP group in terms of intraoperative blood loss (47.0 ± 60.4 vs. 87.1 ± 101.2 ml, P = 0.011) and notch length (1.3 ± 0.4 vs. 6.5 ± 0.8 cm, P = 0.001) (Table 2). Moreover, the NOSES group also fared better in terms of postoperative pain scores (P < 0.001) and the use of additional analgesics (36.8% vs. 52.8%, P = 0.019) (Table 2 and Fig. 2A). There was no significant difference in the detection rate of lymph nodes and the positive rate of upper and lower resection margins between the two groups. In the NOSES group, When we looked at the postoperative recovery, we found that the NOSES group had significantly fewer postoperative complications were observed than that in the LAP group (5.7% vs. 15.5%, P = 0.020). Moreover, the recovery time of gastrointestinal function (50.7 ± 27.3 vs. 58.6 ± 28.5 h, P = 0.040) and the length of total hospital stays (10.2 ± 6.6 vs. 13.0 ± 4.3 days, P < 0.001) in NOSES group were significantly shorter than those in LAP group (Table 2). We can see that there is no significant difference in PFDI-20 between the two groups postoperatively (Table 2). We found that patients in the NOSES group had significantly better physiological function (P = 0.003), role function (P < 0.001), emotional function (P < 0.001), cognitive function (P = 0.046) and global health status (P = 0.008) than those in the LAP group. Moreover, patients in the NOSES group had less pain (P < 0.001), insomnia (P < 0.001), constipation (P < 0.001) and diarrhea (P < 0.001) (Fig. 2B,C). Patients in the noses group reported greater satisfaction with body image (P = 0.001) and cosmesis (P < 0.001) postoperatively (Fig. 2D). And there was no significant difference in anal function between the two groups (Table 3). These results indicated that NOSES may have a better short-term outcomes than traditional laparoscopic surgery in rectal cancer.

**Table 2:** Comparison of postoperative conditions between the two groups

Outcome	After PSM			
	LAP (N = 106)	NOSES (N = 106)	t/χ^2^	P value
Operative time (min)	183.9 ± 51.7	187.2 ± 50.5	− 0.471	0.638
Blood loss (mL)	87.1 ± 101.2	47.0 ± 60.4	2.541	0.011
Notch length^a^ (cm)	6.5 ± 0.8	1.3 ± 0.4	3.502	0.001
Harvested lymph node (pieces)	13.6 ± 5.0	13.7 ± 5.8	− 0.102	0.919
Positive lymph node (pieces)	1.1 ± 2.6	0.7 ± 1.6	1.427	0.155
Positive margin	0 (0)	0 (0)	NA	NA
Intraoperative complications	0 (0)	0 (0)	NA	NA
Grade			2.912	0.233
Well-differentiated	23 (21.7%)	23 (21.7%)		
Moderately-differentiated	77 (72.6%)	70 (66.0%)		
Poor-differentiated	6 (5.7%)	13 (12.3%)		
Histology			0.712	0.701
Adenocarcinoma	100 (94.3%)	99 (93.4%)		
Tubular adenocarcinoma	3 (2.8%)	2 (1.9%)		
Mucinous	3 (2.8%)	5 (4.7%)		
Usage of additional analgesics	56 (52.8%)	39 (36.8%)	5.512	0.019
VAS scores			/	< 0.001*
Day 1 postoperatively	4.16 ± 1.36	3.29 ± 1.32		
Day 3 postoperatively	3.29 ± 1.13	2.46 ± 1.17		
Day 5 postoperatively	1.93 ± 0.78	1.53 ± 0.72		
Time to recovery of gastrointestinal function (hour)	58.6 ± 28.5	50.7 ± 27.3	2.065	0.040
Length of total hospital stays (day)	13.0 ± 4.3	10.2 ± 6.6	3.713	< 0.001
Postoperative complication	16 (15.5%)	6 (5.7%)	5.407	0.020
Anastomotic leakage	1 (0.9%)	2 (1.9%)		
Intra-abdominal infection	1 (0.9%)	0 (0%)		
Ileus	1 (0.9%)	0 (0%)		
Pneumonia	2 (1.9%)	1 (0.9%)		
Incision-related complications	11 (10.4%)	3 (2.8%)		
Pulmonary embolism	0 (0%)	0 (0%)		
Postoperative PFDI-20 scores	6.47 ± 1.87	6.56 ± 1.35	− 0.378	0.706
Medical expenses (RMB)	71,280 ± 17,870	72,085 ± 20,265	− 0.306	0760

Results in the table are presented as mean ± standard deviation or number (%); a: The notch length was considered to be the length at which the abdominal wall was maximally incised

*LAP* conventional laparoscopic assisted resection group, *NOSES*, natural orifice specimen extraction surgery group, *PSM* propensity score matching, *VAS scores* visual analogue scale scores, *PFDI-20* Pelvic Floor Distress Inventory-20

*The P-value was calculated by repeated measures statistical analysis

**Table 3:** Postoperative Wexner scores in both groups

Type of Incontinence	After PSM			
	LAP (N = 106)	NOSES (N = 186)	Z	P value
Solid	2.23	2.44	− 1.548	0.122
Liquid	1.18	2.00	− 1.018	0.309
Gas	2.39	2.01	− 1.960	0.050
Wears pad	0.43	0.54	− 1.015	0.310
Lifestyle alteration	2.11	1.75	− 2.587	0.010

Results in the table are presented as mean

*LAP* conventional laparoscopic assisted resection group, *NOSES*, natural orifice specimen extraction surgery group, *PSM* propensity score matching

### Comparison of postoperative survival status of the two groups

**Fig. 3:**
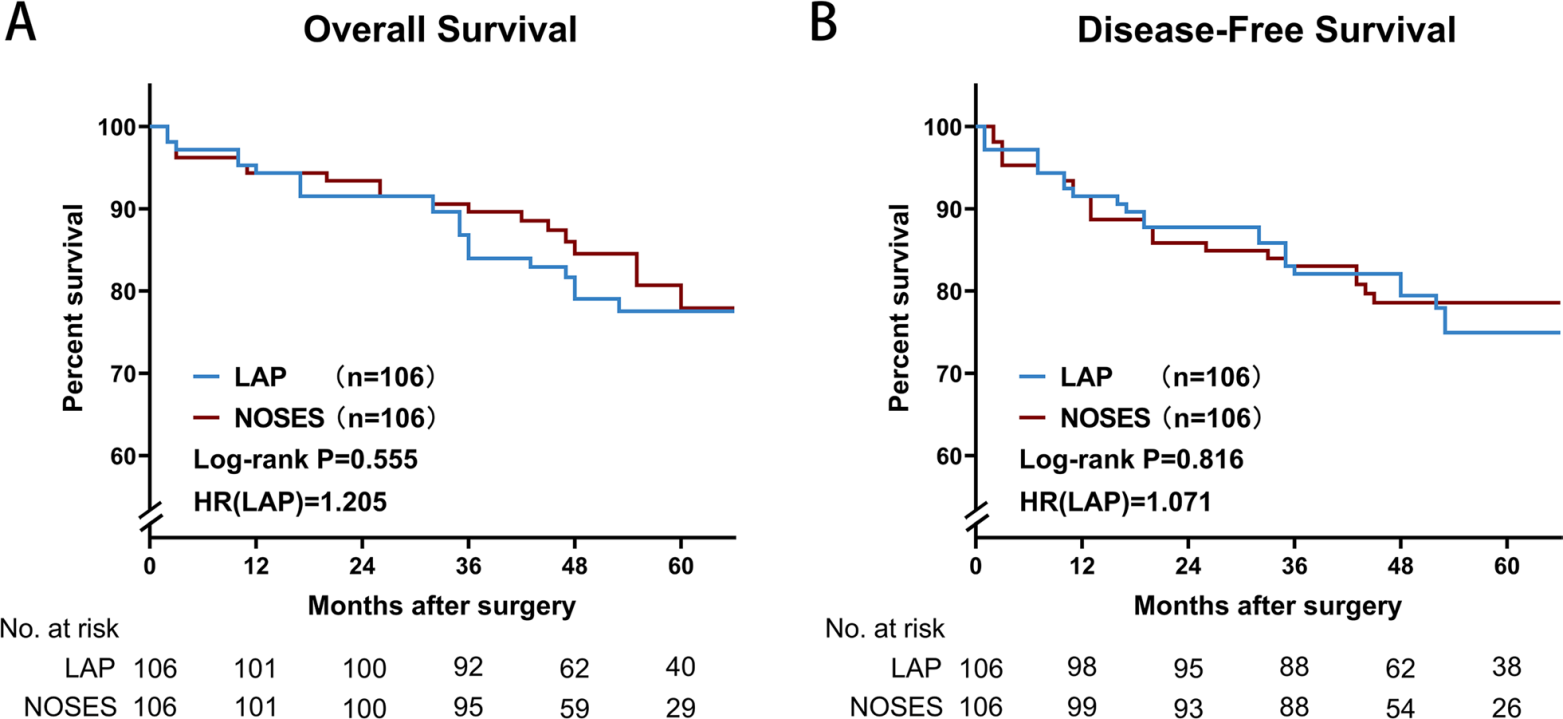
Comparison of long-term curative effect between two groups of patients. **A** Overall survival, **B** Disease-free survival

The most concerned thing about a new procedure is whether it has a negative impact on the long-term survival of the patient. There was no significant difference in overall survival (OS) and disease-free survival (DFS) between the two groups (Fig. 3). There was no significant difference in recurrence and metastasis between the two groups (Table 4).

**Table 4:** Comparison of the long-term outcomes of the two groups

Outcome	Before PSM			
	LAP (N = 106)	NOSES (N = 106)	χ^2^	P value
Status at the last follow-up (N%)			0.493	0.483
Survival	84 (79.2%)	88 (83.0%)		
Dead	22 (20.8%)	18 (17.0%)		
Local recurrence (N%)	6 (5.7%)	7 (6.6%)	0.082	0.775
Distant metastasis (N%)	8 (7.5%)	8 (7.5%)	0.000	1.00
Liver metastasis	5 (4.7%)	4 (3.8%)		/
Lung metastasis	3 (2.8%)	4 (3.8%)		/

*LAP* conventional laparoscopic assisted resection group, *NOSES*, natural orifice specimen extraction surgery group, *PSM* propensity score matching

## Discussion

Colorectal cancer is a serious threat to public health, and surgical treatment has become the first option [17]. Laparoscopic radical resection for rectal cancer has been widely used, but the assisted incision in the abdominal wall could induce great trauma to the patients, which was not conducive to the postoperative recovery of the patient [18, 19]. In recent years, with the development of minimally invasive technique, NOSES has become one of the most popular producers, which has the advantages of less trauma, shorter scar, shorter healing time, and fewer complications [20, 21]. The results of this study showed that intraoperative blood loss, additional analgesic use, postoperative gastrointestinal recovery time, and hospital stay in the NOSES group were better than those in the conventional laparoscopic group.

The current focus of NOSES is on the safety of the procedure, that is, aseptic principle and tumor free principle. Transrectal or transvaginal incision remain the major challenge, which may lead to implantation and cancer spread, or intestinal bacteria or entering the abdominal cavity through rectal or vagina. However, peritoneal contamination and tumor implantation can be effectively avoided by adequate preoperative preparation, rational use of antineoplastic drugs and antibiotics, application of protective sleeve and extensive sterile irrigation [11]. In our study, there was no difference in the incidence of intra-postoperative abdominal infection and tumor recurrence rates. Intraoperative lymph node dissection also significantly affected patients’ outcomes, whereas the average numbers of lymph nodes harvested by both producers in this study were more than twelve, consistent with the principle of radicality [22]. There was no significant difference between the two groups in terms of tumor recurrence and metastasis, or long-term survival. The safety of The use of sterile gloves, adequate lymph node dissection and strict adherence to the aseptic principle and the tumor free principle ensured the safety of NOSES.

In the NOSES group, intraoperative bleeding was less due to the fact that NOSES usually requires more detailed dissection, complete exposure of the colorectum, and the use of Hom-o-Lok clips to clamp all of pre-disconnected vessels to secure accidental bleeding [23]. In addition, NOSES is performed intra-abdominally throughout the procedure, without the need to incise the abdominal wall, pull out the rectum and rectal mesentery through the incision and deal with part of the mesentery outside the abdominal cavity, which can greatly reduce intraoperative bleeding due to violent operations. In the postoperative period, the patients were encouraged to cough, expectorate and move out of bed to prevent pneumonia and other respiratory infections [24]. Patients who underwent NOSES had no incision on the abdominal wall, and they were more willing to cough and expectorate because they had less pain and psychological concerns. The patients underwent NOSES could move as early as they can, promoting recovery of gastrointestinal function, expedite the time of first flatus after surgery; thus, functional recovery become better and faster [8, 25]. In addition, the operation of NOSES is carried out completely in the abdominal cavity, avoiding the contact of abdominal organs with the outside world, reducing the stimulation of abdominal organs, and speeding up the recovery. The quality of life in NOSES group was significantly better than that in LAP group, which is an indication of the overall recovery.

Postoperative complications are also an important index to evaluate the safety of operation. And postoperative complications not only prolong the length of hospital stay and increase the costs, but also reduce the patient-physician trust [26]. We thought that the higher complication rate of LAP group is due to the increased incidence of abdominal wound complications. In contrast, NOSES have no auxiliary incision, resulting in fewer complications [27].

For rectal cancer patients undergoing surgery, the long-term outcome of the surgery has a direct impact on survival. There was no significant difference in recurrence, distant metastasis, OS and DFS between the two groups. These results suggest that the NOSES radical resection of rectal cancer can achieve the same long-term results as laparoscopic resection of rectal cancer. Nevertheless, we should still strictly evaluate the patient’s condition and surgical indications due to the limitation of NOSES to the size and location of the tumor, in order to avoid surgical failure [8].

The authors acknowledges that there are certain limitations in this study. First of all, we cannot avoid the bias in surgical research, such as different surgeons. However, we have minimized the bias. All doctors who perform conventional laparoscopic and NOSES radical rectal cancer surgery have undergone sufficient clinical practice. Everyone has completed at least 100 cases of laparoscopic radical rectal cancer surgery and has sufficient surgical experience and skills. Furthermore, this study is a retrospective study, and selection bias is inevitable. Thus, we used propensity score matching to minimize the difference in baseline information between the two groups.

According to our current results, NOSES is superior to conventional laparoscopy in terms of short-term efficacy, and there is no significant difference in long-term survival compared with conventional laparoscopy. We look forward to more prospective studies to explore the difference between NOSES and conventional laparoscopy surgery.

## Supplementary Information

**Additional file 1:** Body Image Questionnaire.

## Data Availability

The datasets generated and analyzed during the current study are not publicly available due to the confidentiality requirements of research institutions but are available from the corresponding author on reasonable request.
