# Supplementary material for: Long-term oncologic outcomes of natural orifice specimen extraction surgery versus conventional laparoscopic-assisted resection in the treatment of rectal cancer: a propensity-score matching study
Source: BMC Surg. 2022 Jul 25;22:286. doi: 10.1186/s12893-022-01737-2 (PMC9317461; doi:10.1186/s12893-022-01737-2)
Supplement: Supplementary file 1 — Additional file 1: Body Image Questionnaire. [file 12893_2022_1737_MOESM1_ESM.docx]

Table S1: Wexner Incontinence Score

| Type of Incontinence | Frequency | | | | |
| --- | --- | --- | --- | --- | --- |
|  | Never | Rarely | Sometimes | Usually | Always |
| Solid | 0 | 1 | 2 | 3 | 4 |
| Liquid | 0 | 1 | 2 | 3 | 4 |
| Gas | 0 | 1 | 2 | 3 | 4 |
| Wears pad | 0 | 1 | 2 | 3 | 4 |
| Lifestyle alteration | 0 | 1 | 2 | 3 | 4 |

**Notes:**

1. 0 = perfect. 20 = completely incontinence.

2. Never = 0 (never); Rarely, ≤ 1/month; Sometimes, ≤ 1/week, ≥ 1/month; Usually, ≤ 1/day, ≥ 1/week; Always ≥ 1/day.

Additional material 1:

**Body Image Questionnaire**

1. Are you less satisfied with your body since the operation?

range: 1=no, not at all

2= a little bit

3= quite a bit

4= yes, extremely

2. Do you think the operation has damaged your body?

range: 1=no, not at all

2= a little bit

3= quite a bit

4= yes, extremely

3. Do you feel less attractive as a result of your disease or treatment?

range: 1=no, not at all

2= a little bit

3= quite a bit

4= yes, extremely

4. Do you feel less feminine/masculine as a result of your disease or treatment?

range: 1=no, not at all

2= a little bit

3= quite a bit

4= yes, extremely

5. Is it difficult to look at yourself naked?

range: 1=no, not at all

2= a little bit

3= quite a bit

4= yes, extremely

6. On a scale from 1 to 7, how satisfied are you with your (incisional) scar?

| 1= very unsatisfied | 2 | 3 | 4= not unsatisfied / not satisfied | 5 | 6 | 7= very satisfied |
| --- | --- | --- | --- | --- | --- | --- |

7. On a scale from 1 to 7, how would you describe your (incisional) scar?

| 1= revolting | 2 | 3 | 4= not revolting / not beautiful | 5 | 6 | 7= beautiful |
| --- | --- | --- | --- | --- | --- | --- |

8. Could you score your own incisional scar on a scale from 1 to 10?
